# Local anesthesia with sedation and general anesthesia for the treatment of chronic subdural hematoma: a systematic review and meta-analysis

**DOI:** 10.1007/s10143-024-02420-1

**Published:** 2024-04-16

**Authors:** Mariam Ahmed Abdelhady, Ahmed Aljabali, Mohammad Al-Jafari, Ibrahim Serag, Amr Elrosasy, Ahmed Atia, Aya Ehab, Shrouk F. Mohammed, Ibraheem M. Alkhawaldeh, Mohamed Abouzid

**Affiliations:** 1 Faculty of Medicine, October 6 University, Giza, Egypt; 2 Medical Research Group of Egypt, Negida Academy, Arlington, MA USA; 3 Faculty of Medicine, Jordan University of Science and Technology, Irbid, Jordan; 4 Faculty of Medicine, Mutah University, Al-Karak, Jordan; 5 Faculty of Medicine, Mansoura University, Mansoura, Egypt; 6 Faculty of Medicine, Cairo University, Cairo, Egypt; 7 Faculty of Medicine, Aswan University, Aswan, Egypt; 8 Faculty of Medicine, Alexandria University, Alexandria, Egypt; 9 Department of Physical Pharmacy and Pharmacokinetics, Faculty of Pharmacy, Poznan University of Medical Sciences, Rokietnicka 3 St., 60-806 Poznan, Poland; 10 Doctoral School, Poznan University of Medical Sciences, 60-812 Poznan, Poland

**Keywords:** Local anesthesia, General anesthesia, Sedation, Chronic subdural hematoma, Systematic review

## Abstract

**Background:**

Surgery is the primary treatment for chronic subdural hematoma, and anesthesia significantly impacts the surgery's outcomes. A previous systematic review compared general anesthesia to local anesthesia in 319 patients. Our study builds upon this research, analyzing 4,367 cases to provide updated and rigorous evidence.

**Methods:**

We systematically searched five electronic databases: PubMed, Cochrane Library, Scopus, Ovid Medline, and Web of Science, to identify eligible comparative studies. All studies published until September 2023 were included in our analysis. We compared six primary outcomes between the two groups using Review Manager Software.

**Results:**

Eighteen studies involving a total of 4,367 participants were included in the meta-analysis. The analysis revealed no significant difference between the two techniques in terms of 'recurrence rate' (OR = 0.95, 95% CI [0.78 to 1.15], *P* = 0.59), 'mortality rate' (OR = 1.02, 95% CI [0.55 to 1.88], *P* = 0.96), and 'reoperation rate' (OR = 0.95, 95% CI [0.5 to 1.79], *P* = 0.87). Local anesthesia demonstrated superiority with a lower 'complications rate' than general anesthesia, as the latter had almost 2.4 times higher odds of experiencing complications (OR = 2.4, 95% CI [1.81 to 3.17], *P* < 0.00001). Additionally, local anesthesia was associated with a shorter 'length of hospital stay' (SMD = 1.19, 95% CI [1.06 to 1.32], *P* < 0.00001) and a reduced 'duration of surgery' (SMD = 0.94, 95% CI [0.67 to 1.2], *P* < 0.00001).

**Conclusion:**

Surgery for chronic subdural hematoma under local anesthesia results in fewer complications, a shorter length of hospital stay, and a shorter duration of the operation.

## Introduction

Chronic subdural hematoma (CSDH) is one of the most common pathologies in the neurosurgical field; it affects 1.7–20.6 per 100,000 individuals per year, especially the elderly in their 9^th^ decade [1–3]. The pathophysiology of CSDH involves a sequence of head trauma, inflammation, an aberrant cascade of coagulopathy, angiogenesis, recurrent microhemorrhages, and exudates. The mechanism of CSDH associated with spontaneous intracranial hypotension consists of a decrease in cerebrospinal fluid pressure, leading to downward displacement of the brain. This displacement can result in venous stretching and tearing, causing bleeding and accumulation of blood in the subdural space, resulting in hematoma formation. The low CSF pressure also contributes to the failure of the hematoma to reabsorb naturally [4]

Serial neurologic examinations and imaging studies can follow patients with mild symptoms. Suggested medications for conservative medications include atorvastatin, dexamethasone, and tranexamic acid. A study conducted by Wang et al. [5] revealed that dexamethasone and atorvastatin effectively reduce CSDH recurrence, but dexamethasone also increases mortality risk. Atorvastatin is preferred for reducing hematoma volume, and dexamethasone is the leading option for treating CSDH, but we should use dexamethasone with caution due to its risks [6, 7]. However, the treatment options do not only depend on the severity of symptoms but also on their dynamic progression and computed tomography imaging data. Therefore, patients with evident symptoms and progressive worsening of the neurological status and imaging evidence of significant cerebral shift are treated surgically using burr hole craniostomy, drainage of the hematoma, craniotomy, and endovascular obliteration of the middle meningeal artery, which seems to be the most frequently used surgical evacuation procedure [2, 8]. We have discussed earlier the impact of drainage and irrigation in the treatment of CSDH [9, 10].

CSDH evacuation procedures such as Burr hole craniostomy are done under local anesthesia (LA) or general anesthesia (GA). Local anesthesia is safer and reduces the risk of serious complications such as aspiration pneumonia, thrombosis, and hemodynamic instability, which may occur with the GA. However, LA is not ideal with agitated or uncooperative patients, so it can be combined with sedatives such as dexmedetomidine, midazolam, propofol, or opioids to prevent the intra-operative and postoperative complications of GA while achieving appropriate patient compliance [11–14].

Two clinical trials [12, 15] compared LA and GA during the evacuation of CSDH in terms of intra-operative and postoperative complications like hemodynamic fluctuations, operative time, and length of hospital stay. A meta-analysis [16] evaluated the medical effectiveness of the advocated anesthetic techniques. This study aims to update the most recent literature and provide a robust analysis evaluating the best anesthesia technique for CSDH.

## Methods

We followed the PRISMA statement guidelines for this systematic review and meta-analysis [17].

### Eligibility criteria

This research involved studies that met the following criteria:

1. randomized controlled trials, non-randomized controlled trials, and observational studies
2. studies whose populations were chronic subdural hematoma patients
3. studies that considered general anesthesia as an intervention
4. studies that considered local anesthesia as a comparator
5. studies that report at least one of the following outcomes: recurrence, complications, mortality, reoperation, hospital stay, and operation length.

We excluded animal studies, case series, case reports, theses, and secondary analysis studies; conference abstracts; editorial letters; studies that lack a comparator; and studies whose data extraction and analysis were unreliable.

### Search strategy and selection of studies

We conducted our search using the following electronic databases through September 2023: PubMed, Scopus, the Cochrane Central Register of Controlled Trials, Ovid Medline, and Web of Science, using the following query: (chronic subdural hematoma) OR (CSDH) OR (subdural hematoma) OR (subdural hemorrhage) OR (subdural bleeding) AND (local anesthesia) AND (general anesthesia OR anesthesia OR sedation).

After removing duplicate studies from the found records, three authors (A.E, A.N, and A.E) checked each study for eligibility in two steps. The first step was to determine eligibility by screening titles and abstracts. In the second stage, the full-text articles of suitable abstracts were retrieved and screened. Rayyan software package was used for this approach [18].

### Data extraction

Two authors (A.E and S.F.M) independently extracted the data using an online data extraction form. The extracted data included the following: (1) study characteristics, (2) characteristics of the study population, (3) risk of bias domains, and (4) study outcomes.

### Statistical analysis and heterogeneity

We used RevMan 5.3 software (Cochrane, London, UK) to perform the analysis. Changes in dichotomous variables (recurrence, complications, mortality, and reoperation) were pooled as odds ratios (OR) via the Mantel–Haenzel (M–H) method. Changes in continuous variables (length of hospital stay and length of operation) were pooled as a mean difference (MD). We adopted the random effects model because it is based on the assumption that studies represent a random sample of the population. This model is characterized by a wider standard error, a larger weight to smaller studies, and a wider confidence interval. When data were reported as median Inter Quartile Range (IQR), we converted it to mean (SD), According to Wan's formula [19]. In the absence of heterogeneity, a fixed effects model with the assumption that effect size is constant across trials was adopted.

Visual assessment of the forest plots was used to determine heterogeneity, and the I^2^ and chi-square (χ2) tests were used to measure it. The presence of notable heterogeneity was investigated using the χ2 test, and if heterogeneity was found, it was quantified using the I^2^ test. The Cochrane Handbook's guidelines for meta-analysis were followed when interpreting the I^2^ test (0–40% = may not be significant, 30–60% = may represent moderate heterogeneity, 50–90% = may represent substantial heterogeneity, and 75–100% = significant heterogeneity).

The pooled effect estimate was plotted against its SE in a funnel plot generated by the RevMan program to assess publication bias. The degree of the figure symmetry was used to establish whether or not publication bias existed. Also, according to Egger and colleagues [20, 21], evaluating publication bias is valid for > 10 pooled studies. As a result, in this work, we adopted Egger's test for funnel plot asymmetry to determine the presence of publication bias.

### Quality assessment

The Cochrane risk of bias (ROB) tool was used to assess the quality of RCTs, whereas the Newcastle–Ottawa Scale (NOS) was used to assess the quality of observational studies [22, 23].

### Sensitivity analysis

We ran a sensitivity analysis to investigate any considerable heterogeneity detected in outcomes.

## Results

### Literature search

**Fig. 1:**
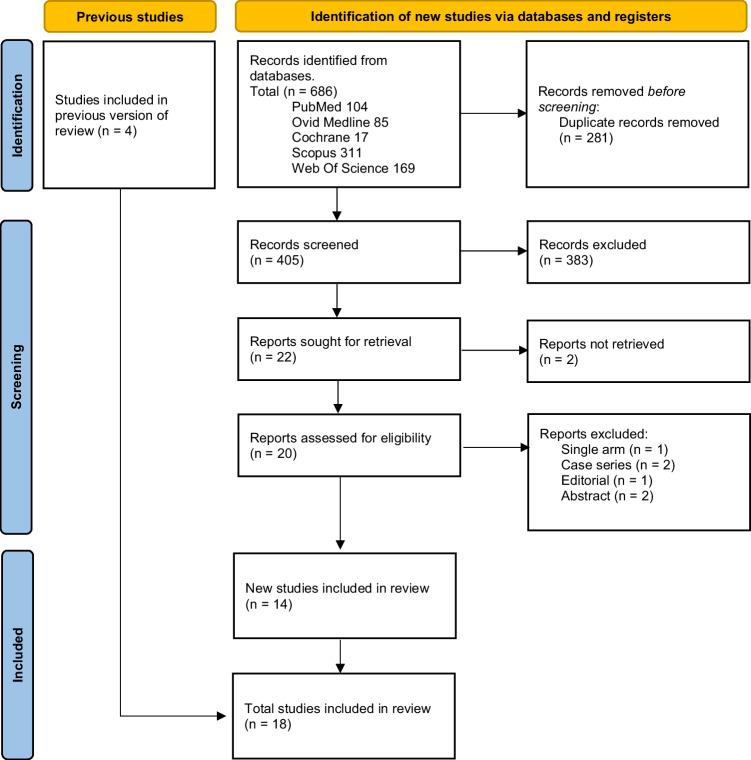
Description of the study selection process in coherence with the Preferred Reporting Items for Systematic Reviews and Meta-analyses (PRISMA) guidelines

Figure 1 displays a flow chart of papers selected and included following PRISMA standards [17]. An electronic search of databases identified 686 records; 405 were included in the title and abstract screening, and the remaining 281 were duplicates; 383 were excluded as they did not meet our inclusion criteria. We conducted the full-text screening on the eligible 22 studies. By full-text screening, 18 studies with 4,367 patients met our inclusion criteria and were included in the present analysis.

### Characteristics of the included studies

The included studies' summary and patients' baseline characteristics are shown in (Table 1). There were 14 retrospective cohort studies, two randomized clinical trials, and two case controls. The most extensive study included 923 patients (314 GA and 609 LA), while the smallest included only 30 (15 GA and 15 LA). The mean age of the patients ranged from 58 to 76 years in the included studies, varying among 1425 females and 4004 males in all included studies. All studies conducted the CSDH drainage surgery using single or double burr hole techniques.

**Table 1:** Baseline characteristics for included studies

Ref	Study ID	Age, mean (SD)	Sex		Study design	Country	Surgical method	Sample (n)			Follow up	GCS	
		GA	LA	F: M				GA	LA	Total		GA	LA
[24]	**Alnaami 2021**	63.55 (20.58)	-	18:11	Case series	Saudi Arabia	CSDH-related drainage surgery	47	41	88	6 m	-	-
[25]	**Blaauw 2020**	73.5 (11)	-	237:36	Retrospective cohort	Netherlands	Burr-hole drainage with irrigation	314	609	923	-	15 (1)	
[26]	**Chen 2020**	68.1 (12.4)	-	11:19	Retrospective cohort	Italy	Single-burr hole technique for subdural drainage	15	15	30	18.2 (range 10–29) in GA and 15.2 (range 8–28) in LA	-	-
[27]	**Francesco certo 2019**	68.1 (12.4)	-	99:54	Retrospective cohort	China	Single burr hole	247	201	448	12 m	-	-
[28]	**Gelabert 2015**	74.1 (14.67)	-	543:36	Retrospective cohort	Spain	One or two burr holes surgery	39	151	190	-	-	-
[29]	**Han 2017**	67.9 (8.3)	-	191:34	Retrospective cohort	Korea	Standard 1– or 2–bur hole craniotomy	207	549	756	6 m	-	-
[15]	**Hestin 2022**	75 (9)	76 (11)	17:43	RCT	France	Craniotomy or burr hole craniotomy	30	30	60	-	-	-
[30]	**Iftikhar 2016**	64.5 (13.5)	-	11:45	Retrospective chart review	Pakistan	Burr hole surgery with irrigation, or without irrigation	31	25	56	> 1 m, < 1 year	15	15
[31]	**Katsuki 2020**	85.67 (23.96)	-	14:21	Case series-case control	Japan	Endoscopic technique for subdural drainage	0	35	35	-	10.67 (9.28)	
[32]	**Kostas 2019**	-	-	53	Retrospective cohort	Greece	Burr-hole drainage	125	46	171	> 3 m	-	-
[33]	**Shaikh Mahmood 2017**	69.75 (20.02)	68.67 (23.57)	10:25	Retrospective chart review	Pakistan	Craniotomy with single burr hole	19	16	35	In GA median of 3 m, LA median of 4 m	10 (9.6)	12.3 (6.5)
[34]	**Shen 2019**	-	-	66:40	Retrospective cohort	China	Craniotomy with single burr hole	212	130	342	> 3 m	-	-
[35]	**Shen B 2019**	-	-	87:16	Retrospective cohort	China	Craniotomy with single burr hole	307	164	457	> 3 m	-	-
[12]	**Surve Rohini 2016**	58.79 (14.97)	57.63 (15.08)	8:05	RCT	India	Craniotomy with single burr hole	34	38	76	-	13.85 (1.76)	14.03 (1.48)
[36]	**Wong 2022**	69.5 (12.4)	66.8 (14.3)	59:21	Retrospective cohort	United Kingdom	Craniotomy with single burr hole	127	130	257	LA 11 m, GA 4.3 m	-	-
[37]	**Zhuang 2022**	66.63 (12.41)	62.25 (11.54)	15:31	Retrospective cohort	China	Craniotomy with single burr hole	54	51	105	> 1 m, < 1 year	14.89 (0.42)	14.90 (0.36)
[29]	**Jin Oh 2022**	-	-	85:208	Retrospective cohort	Korea	craniotomy with single burr hole	206	87	293	126.2 days (range, 1–807)	-	-

*RCT* randomized control trial, *GA* general anesthesia, *LA* local anesthesia, *SD* standard deviation, *NR* not mentioned, *GCS* glasgow coma scale, *m* month

### Quality assessment

**Fig. 2:**
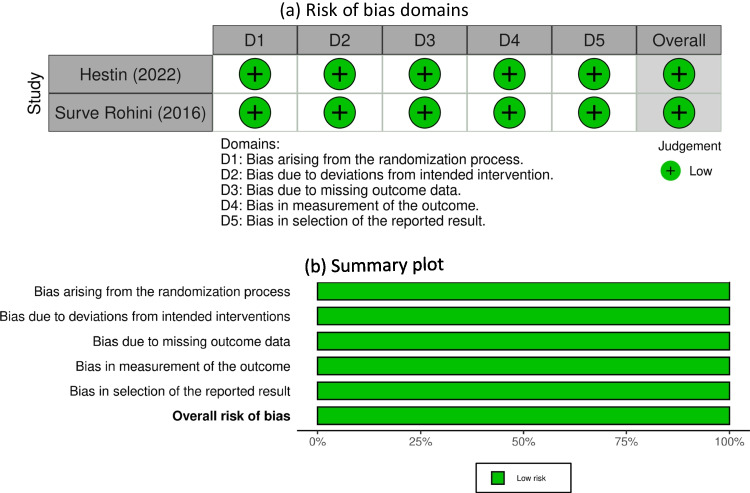
(**a**) Risk of bias domains; (**b**) Summary of the plot for the included RCTs

**Fig. 3:**
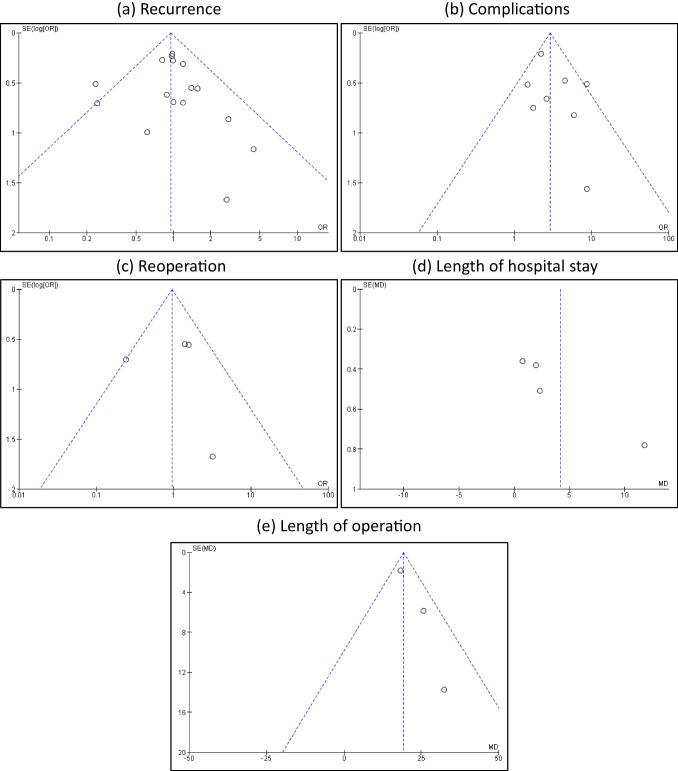
A funnel plot was used to assess publication bias in studies reporting (**a**) recurrence; (**b**) complications; (**c**) reoperation; (**d**) length of hospital stay; and (**e**) length of operation

The selected studies ranged in quality from moderate to high, according to the Risk of Bias (RoB-2) tool for randomized controlled trials and the modified Newcastle Ottawa scale (NOS) assessment tool for observational studies (Tables 2, 3, and Fig. 2). Moreover, we also did not notice significant bias according to Egger's test for recurrence rate (n = 16 studies) (intercept (B_0_) 0.4, 95% CI [-0.51, 1.32], *P* = 0.35). A funnel plot was used to assess publication bias in studies shown in (Fig. 3).

**Table 2:** The Newcastle–Ottawa Scale (NOS) quality assessment for case-controlled studies

Study ID	Case definition Adequate	Representativeness of the cases	Selection of Controls	Definition of Controls	Comparability based on design or analysis part 1	Comparability based on design or analysis part 2	Ascertainment of Exposure	Same method of ascertainment for cases and controls	Other	Overall
Francesco certo 2019	Low	High	Low	Low	Low	Low	Low	Low	Unclear	low
Katsuki 2020	Low	High	High	Low	Low	Low	Low	Low	Unclear	Low

**Table 3:** NOS assessing the methodological quality of cohort studies

Study ID	Selection (Max 4)	Comparability (Max 2)	Outcome (Max 3)	Total (Max 9)	Judgment
Alnaami 2021	2	1	2	5	Moderate
Ashry 2022	3	2	2	7	Low
Blaauw 2020	3	2	2	7	Low
Chen 2020	3	1	1	5	Low
Gelabert 2015	4	1	1	6	Moderate
Han 2017	4	2	2	8	Low
Iftikhar 2016	3	1	2	6	Moderate
Kostas 2019	3	1	2	6	Moderate
Shaikh Mahmood 2017	3	2	2	7	Low
Shen 2019	3	1	3	7	Low
Shen B 2019	3	1	3	7	Low
Wong 2022	2	2	2	6	Moderate
Zhuang 2022	3	2	3	8	Low
Jin Oh 2022	2	2	3	7	Low

*NOS* New castle Ottawa scale

### Data analysis

**Fig. 4:**
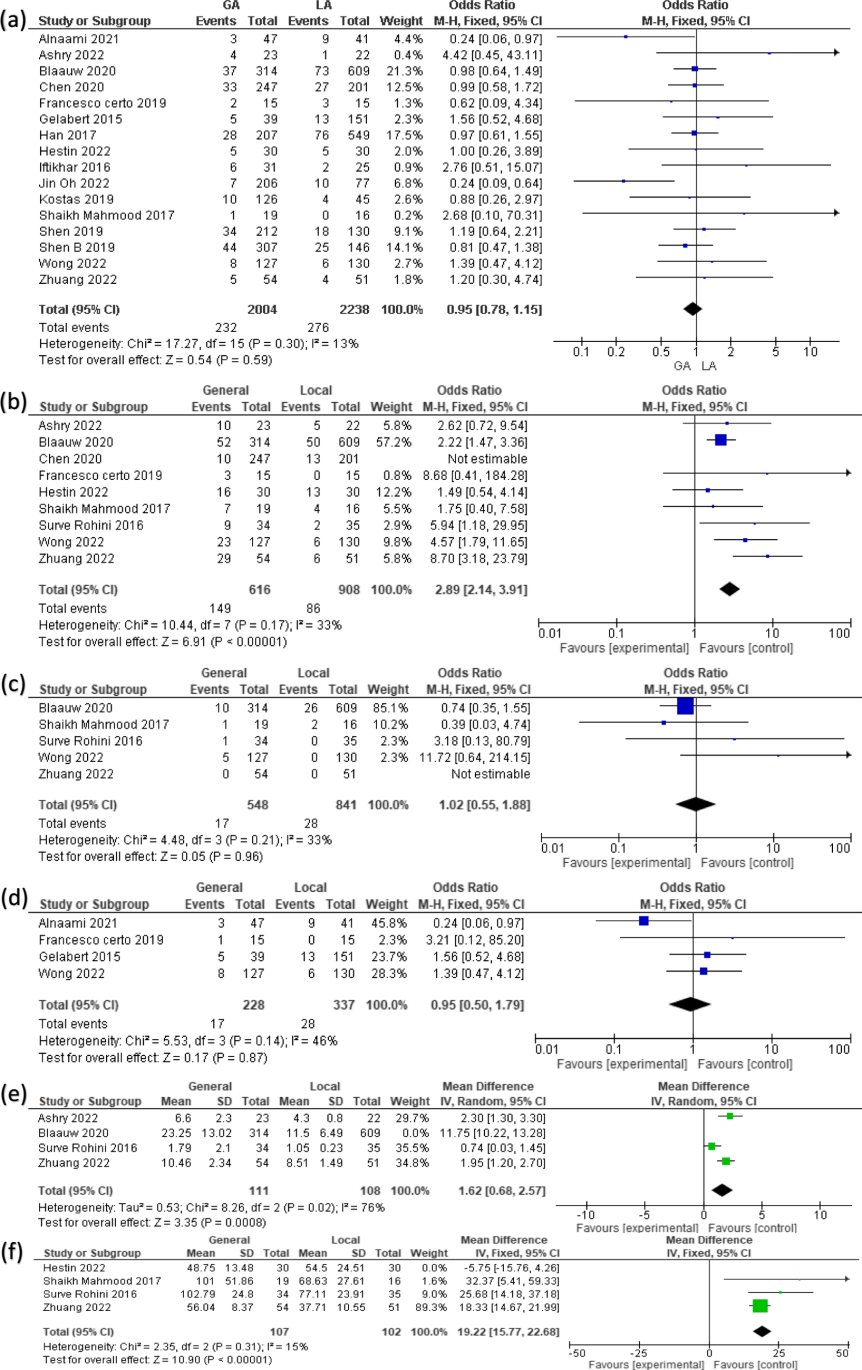
A Forest plot analyzing postoperative (**a**) recurrence; (**b**) complications; (**c**) mortality and (**d**) reoperation after GA and LA. Additionally, it examines the effects of GA and LA on the length of (**e**) hospital stay; and (**f**) operation

There was no significant difference between the GA and LA groups regarding the overall odds ratio of the recurrence rate (OR 0.95, 95% CI [0.78, 1.15], *P* = 0.59). Pooled studies had low heterogeneity (Chi-square *P* = 0.30, I^2^ = 13%) (Fig. 4a).

The overall odds ratio between GA and LA favored using LA over GA. GA has higher complications (OR 2.40, 95% CI [1.81, 3.17], *P* < 0.00001). We detected heterogeneity in this analysis (Chi-square *P* = 0.007, I^2^ = 62%), for which we conducted a sensitivity analysis by the exclusion of (Chen 2020), the result was as follows: (OR 2.89, 95% CI [2.14, 3.91], *P* < 0.00001) (Fig. 4b).

We found no detected statistical difference regarding mortality (OR 1.02, 95% CI [0.55, 1.88], *P* = 0.96). Pooled studies were homogenous (Chi-square *P* = 0.21, I^2^ = 33%) (Fig. 4c).

The overall odds ratio between GA and LA did not favor either of the two groups (OR 0.95 [0.50, 1.79], *P* = 0.87) regarding reoperation. Pooled studies were homogenous (Chi-square *P* = 0.14, I^2^ = 46%) (Fig. 4d).

The length of hospital stay was longer in GA vs LA (MD 4.12, 95% CI [0.72, 7.52], *P* = 0.02). We detected heterogeneity regarding this analysis (Chi-square *P* < 0.00001, I^2^ = 98%). We excluded the study (Blaauw 2020) by sensitivity analysis, the heterogeneity was markedly reduced (Chi-square *P* = 0.02, I^2^ = 76%), and the obtained MD was (1.62, 95% CI [0.68, 2.57], *P* = 0.0008) (Fig. 4e).

The overall mean difference favored LA over GA in terms of length of operation (MD 6.56 [13.30, 19.83], *P* < 0.00001). We detected heterogeneity regarding this analysis (Chi-square *P* < 0.0001, I^2^ = 87%). We excluded the study (Hestin 2022) by sensitivity analysis, and the heterogeneity was resolved. The resulting MD was (19.22, 95% CI [15.77, 22.68], *P* < 0.00001) (Fig. 4f).

## Discussion

Our meta-analysis shows that the LA technique is superior to the GA technique in terms of complication, operation length, and hospital stay. Also, we did not find significant differences between GA and LA patients regarding recurrence rate, mortality, or reoperation.

This meta-analysis results align with those reported by Liu et al. [16] regarding mortality, postoperative recurrence, total duration of surgery, and postoperative complications. Regarding the length of hospital stay, in Liu's study, despite the analyzed studies separately favoring LA, their meta-analysis did not show significant differences. However, our analysis included studies that directly reported the length of stay without conversion and gained significant results in favor of LA. In theory, decreasing the overall duration of surgery should correspondingly reduce the likelihood of surgery-related complications, ultimately leading to shorter hospital stays. Additionally, reducing surgical time will likely decrease demand for post-anesthesia care units. Hence, we noticed that GA length of operation was higher than LA by 19 h (95% CI [15.77, 22.68], *P* < 0.00001). Therefore, LA was associated with a significantly lesser duration of hospital stay than GA, which agrees with previous studies [12, 16, 33, 37]. This is a potential advantage of utilizing LA in the surgical management of CSDH.

It is also worth mentioning that shortening the duration of surgery not only decreases the risk of thromboembolism, hypothermia, and intraoperative adverse events but also eliminates the specific risks associated with GA. Our findings suggested that the GA technique is associated with 2.4 times higher complications compared to the LA technique (95% CI [1.81, 3.17], *P* < 0.00001), similar to previous studies [12, 15, 25, 27, 33, 36–38].

Notably, the causes of death in CSDH may be associated with postoperative complications such as pulmonary infection, thrombosis, and underlying diseases. A retrospective analysis by Wong et al. [22] found that LA significantly reduced the mortality of patients compared with GA. However, regardless of the type of anesthesia, patient death may be associated with underlying diseases such as chronic kidney disease [25, 33]. Our analysis indicated that mortality was not significantly different between LA and GA (*P* = 0.96).

The association between LA and GA and the recurrence rate has been reported previously with conflicting results. Previous studies [24, 27, 32, 35] reported that the LA technique was associated with a significant recurrence rate compared to the GA technique, while other studies showed that the GA technique was associated with a significant recurrence rate compared to the LA technique [28, 30, 33, 34, 36–38]. However, in our meta-analysis, we included 16 studies and noticed an insignificant difference in recurrence between GA and LA (OR 0.95, 95% CI [0.78, 1.15], *P* = 0.59).

Research indicates that the recurrence rate of CSDH post-surgery ranges from 2.5% to 33%, with an increased likelihood in older individuals [39, 40]. The exact causes of relapses remain incompletely understood. Several factors contribute to this risk, including reduced brain tissue elasticity in elderly patients with brain atrophy due to CSDH compression, the persistence of a sizable subdural space post-surgery, the use of antiplatelet medications, stimulation of angiogenesis by growth factors, and inflammatory cytokines. Elevated levels of IL-6 in subdural fluid and factors enhancing the expression of outer membrane VEGF and bFGF also play roles in CSDH recurrence [41]. Effectively managing recurrent CSDH poses a significant challenge, and as highlighted in our previous review, proper drainage after burr-hole evacuation is crucial in mitigating this risk [10]. It is also important to mention that in some studies, recurrence can be defined as exposing the patient to reoperation on the same side [24], while other studies can report the reoperation rate separately. Alnaami et al. study suggested that GA is less associated with reoperation than LA [24], while other studies reported otherwise [27, 28, 36], and the overall analysis of these four studies remained insignificant (*P* = 0.87).

Although surgery for CSDH under results in fewer complications, a shorter hospital stay, and a briefer operation duration, it may not be suitable for all patients. Especially for patients with comorbidities, as described by Certo et al. [27], some individuals with pre-existing neurodegenerative disorders have experienced worsening of their symptoms. Additionally, a patient with Parkinson’s disease exhibited a deterioration in gait disturbances [27]. Generally, in pediatric cases, for instance, LA with sedation can lead to complications such as respiratory depression or atelectasis [42]. Conversely, GA can result in postoperative atelectasis, hemodynamic instability, and aspiration [43]. Therefore, we must choose the type of anesthesia very carefully based on the patient’s specific conditions.

Finally, it is essential to highlight the strengths and limitations of our analysis. To our knowledge, this is the first meta-analysis comprising 18 studies that compare intra-operative and postoperative complications between LA and GA. Among these studies, two were clinical trials, two were case–control studies, and the rest were cohort studies. Additionally, we conducted a rigorous quality assessment, rendering this meta-analysis valuable for clinical physicians in making informed decisions. Furthermore, including studies from various countries worldwide enhances the representativeness of this meta-analysis for the general population.

The limitations of this study include the predominantly observational nature of the research, comprising retrospective and prospective cohort studies, since the operations cannot be conducted blindly. Out of these studies, only two were clinical trials. Additionally, we faced challenges in extracting data from some studies, particularly the mean outcomes, such as the length of hospital stay and the Glasgow Coma Scale, due to unclear information in the papers. Even though we included the bias test for complications, reoperation, length of hospital stay, and length of operation, the number of studies included was less than 10. Hence, the power of this test is low in our analysis, making it difficult to distinguish between chance and real asymmetry. Therefore, the results of Egger’s test should be interpreted with caution.

## Conclusion

No disparities were observed between LA and GA regarding recurrence, mortality, and revision rates. Using LA reduced complications, shorter hospital stays, and operation durations. Therefore, surgeons should individually assess each patient's condition to define the most appropriate treatment plan. We also recommend conducting more clinical trials to thoroughly evaluate the efficacy of LA versus general anesthesia.

## Data Availability

All data generated or analyzed during this study are included in this published article.
